# Shark Fin Occlusive Myocardial Infarction ECG Pattern Post-cardiac Arrest Misinterpreted As Ventricular Tachycardia

**DOI:** 10.7759/cureus.38708

**Published:** 2023-05-08

**Authors:** Jose Escabi-Mendoza, Porfirio E Diaz-Rodriguez, Richard D Silva-Cantillo

**Affiliations:** 1 Cardiology, VA Caribbean Healthcare System, San Juan, PRI

**Keywords:** giant r-wave omi, triangular waveform omi, wide-qrs, lambda, shark-fin, occlusive myocardial infarction, electrocardiogram, stemi, omi, ekg

## Abstract

In addition to the well-known convex ST-segment elevation myocardial infarction (STEMI) pattern associated with acute occlusive myocardial infarction (OMI), there are other cases that are recognized as OMI without fulfilling the established characteristic STEMI criteria. Over one-fourth of the patients initially classified as having non-STEMI can be re-classified as having OMI by recognizing other STEMI equivalent patterns.

We report a case of a 79-year-old man with multiple comorbidities who was brought to the ED by paramedics with a two-hour history of ongoing chest pain. During transport, the patient suffered a cardiac arrest associated with ventricular fibrillation (VF) that required electric defibrillation and active cardiopulmonary resuscitation. Upon ED arrival, the patient was unresponsive, with a heart rate of 150 beats/min and ECG evidence of wide-QRS tachycardia that was misinterpreted as ventricular tachycardia (VT). He was further managed with intravenous amiodarone, mechanical ventilation, sedation, and unsuccessful defibrillation therapy. Upon persistence of the wide-QRS tachycardia and clinical instability, the cardiology team was emergently consulted for bedside assistance. On further review of the ECG, a shark fin (SF) OMI pattern was identified, indicative of an extensive anterolateral OMI. A bedside echocardiogram revealed a severe left ventricular systolic dysfunction with marked anterolateral and apical akinesia. The patient underwent a successful percutaneous coronary intervention (PCI) to an ostial left anterior descending (LAD) culprit occlusion with hemodynamic support but ultimately died due to multiorgan failure and refractory ventricular arrhythmias.

This case illustrates an infrequent OMI presentation (<1.5%) formed by the fusion of the QRS, ST-segment elevation, and T-wave resulting in a wide triangular waveform, giving the appearance of an SF that can also potentially lead to ECG misinterpretation as VT. It also highlights the importance of recognizing STEMI-equivalent ECG patterns to avoid delays in reperfusion therapy. The SF OMI pattern has also been associated with a large amount of ischemic myocardium (such as with left main or proximal LAD occlusion) with a higher mortality risk from cardiogenic shock and/or VF. This high-risk OMI pattern should lead to a more definite reperfusion treatment, such as primary PCI and the possible need for backup hemodynamic support.

## Introduction

In addition to the well-known convex ST-segment elevation myocardial infarction (STEMI) pattern associated with acute occlusive myocardial infarction (OMI), patients may present with uncommon ECG changes such as the shark fin (SF) OMI pattern. This ECG pattern arises due to the merging of the QRS, ST-segment, and T-wave, forming a distinctive wide triangular morphology waveform that can potentially be misinterpreted [1-2] and contribute to reperfusion delay. This article was previously presented as a meeting poster at the Puerto Rican Society of Cardiology in July 2021.

## Case presentation

**Figure 1:**
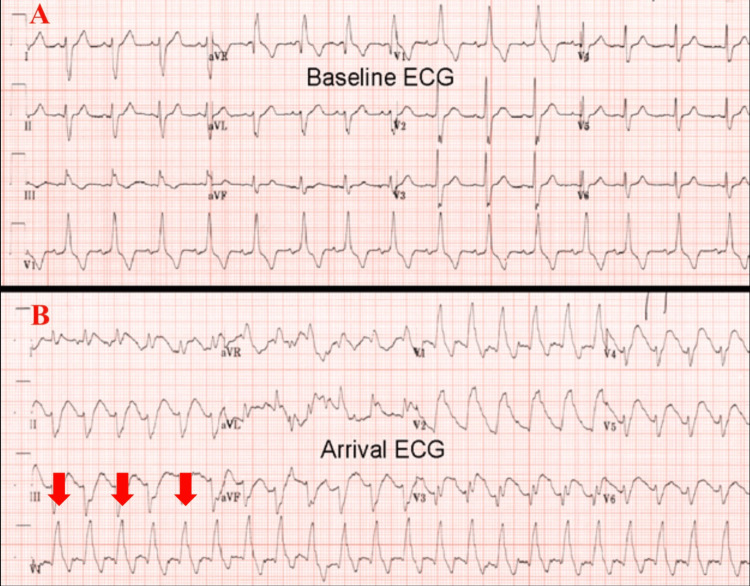
Baseline and arrival ECG A: Baseline ECG. B: Arrival ECG. Red Arrow: wide QRS complex tachycardia

A 79-year-old man with a history of interstitial lung disease, non-obstructive coronary artery disease, psoriasis, and obstructive sleep apnea was brought to the ED by paramedics with ongoing chest pain of two hours of evolution. During transport, the patient suffered a cardiac arrest associated with ventricular fibrillation (VF) that required electric defibrillation and active cardiopulmonary resuscitation. Upon ED arrival, the patient was unresponsive, tachycardic at 150 beats/min, and exhibited a wide-QRS tachycardia on ECG (Figure 1), which was initially misinterpreted as ventricular tachycardia (VT). The patient was administered intravenous amiodarone, placed on mechanical ventilation, sedated, and provided repeat electric defibrillation therapy to resolve the arrhythmia, but these interventions were unsuccessful.

**Figure 2:**
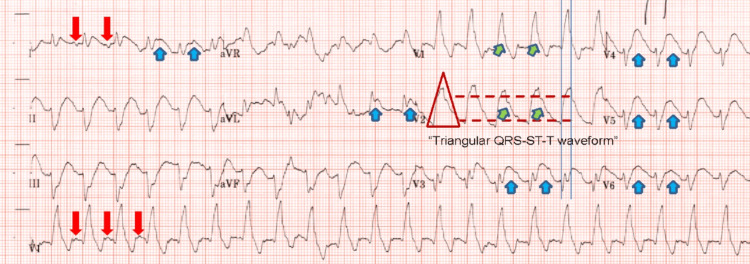
SF pattern STEMI equivalent Red arrows: the presence of P-waves preceding the QRS complexes. Blue arrows: anterior and lateral precordial STEs upon V1-V6, L-1, and aVL, consistent with STEMI. Green arrows: precordial V1 and V2 present with a fusion of QRS, ST-segment, and T-waves giving a triangular waveform, similar to an SF appearance

Upon persistence of the wide-QRS tachycardia and the development of pulmonary edema, the on-call cardiology team was notified for emergent bedside assistance. ECG was thoroughly reviewed and compared with baseline (Figure 1), revealing sinus tachycardia with a chronic right bundle branch block, left anterior fascicular block, and an extensive anterolateral STEMI with anterior and lateral precordial ST-segment elevations (STEs) on leads V1 to V6, I and aVL, and reciprocal ST-segment depression (STD) on leads II, III, and aVF (figure 2). The early precordial leads presented a triangular waveform appearance (in an SF appearance) due to the fusion of the QRS, STE, and T-wave.

**Figure 3:**
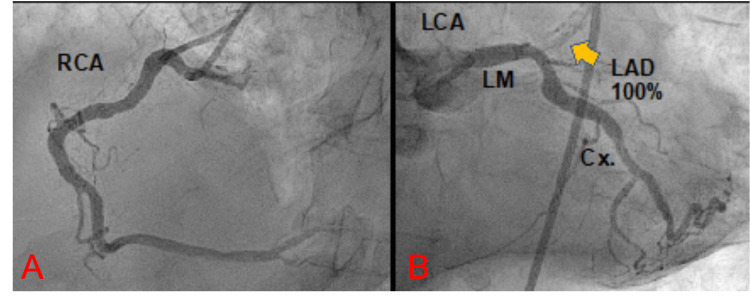
Angiography of the right and left coronary arteries A: angiography of the right coronary artery free of obstructive disease. B: angiography of the left coronary artery showing a 100% occlusion of the LAD artery. Yellow arrow: shows 100% occlusion of the LAD. RCA: right coronary artery. LCA: left coronary artery. LM: left main. LAD: left anterior descending. Cx: circumflex artery

A bedside echocardiogram showed a severe left ventricular systolic dysfunction with marked anterolateral and apical akinesia. The patient was rushed to the Cath-Lab where emergent coronary angiography revealed a 100% occlusion in the ostium of the left anterior descending (LAD) artery (Figure 3).

**Figure 4:**
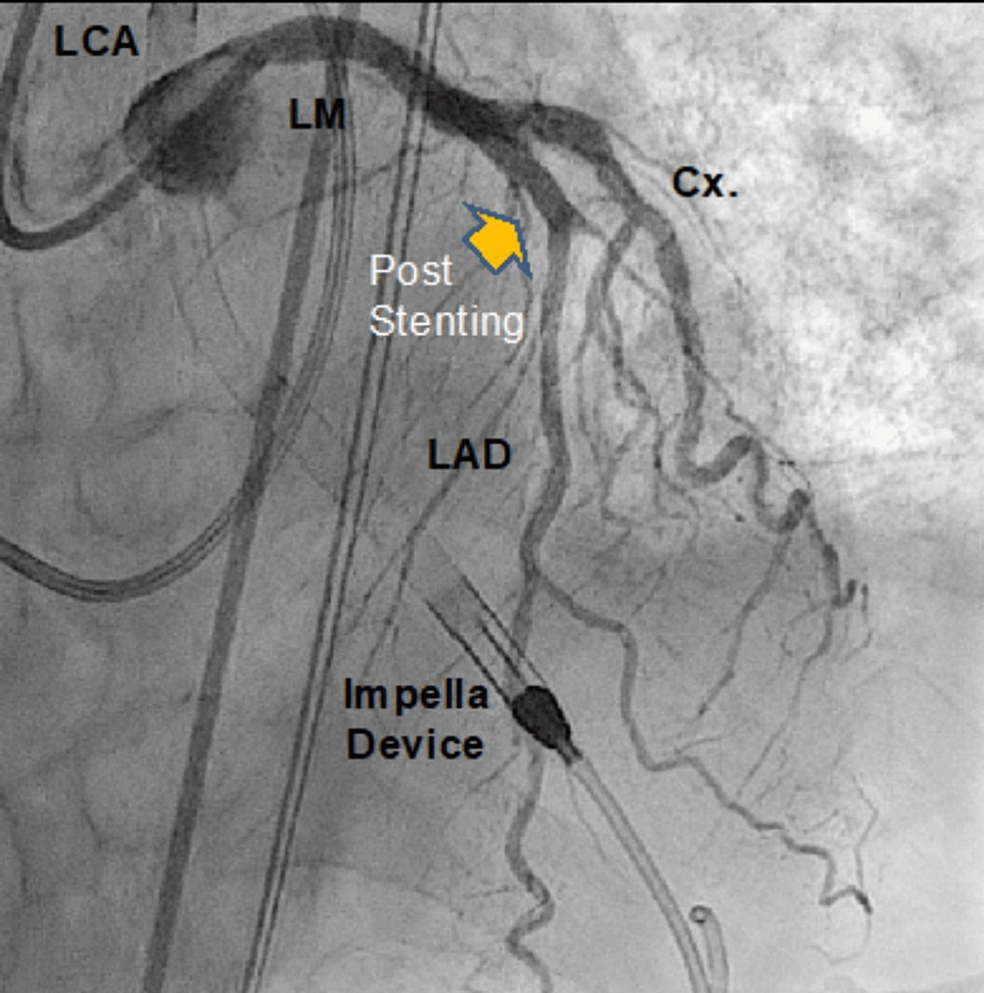
Angiography after successful PCI PCI: percutaneous coronary intervention. LCA: left coronary artery. LM: left main. LAD: left anterior descending. Cx: circumflex artery. Yellow arrow: LAD with flow after stent placement

He underwent a successful PCI stenting under hemodynamic support with an Impella CP assist device, requiring persistent ionotropic and mechanical hemodynamic assistance after the procedure (Figure 4). Despite the above interventions, the patient died from advanced multiorgan failure and refractory ventricular arrhythmias.

## Discussion

Acute coronary occlusive (ACO) myocardial infarction, also known as OMI, is the most serious form of acute myocardial infarction (AMI). ACO is a potentially life-threatening emergency that requires prompt diagnosis and treatment with reperfusion, either fibrinolysis or primary PCI. Despite significant technological advances in many diagnostic fields, the 12-lead ECG remains the cornerstone for the diagnosis and timely reperfusion of an acute OMI.

The paradigm shift of using STE on the ECG as the hallmark for identifying OMI was based on a subgroup analysis of the Fibrinolytic Therapy Trialists' (FTT) meta-analysis [3]. This analysis revealed that the mortality rate was signiﬁcantly lower in patients with STE on ECG who receive fibrinolytic therapy, even in the absence of an angiographic correlate. Subsequently, STEMI became synonymous with OMI, and STEMI criteria were established in the 1996 ACC/AHA guidelines for the management of patients with AMI [4]. After ﬁne-tuning of STE cutoffs, STEMI criteria became and have remained the ECG paradigm universally endorsed in current guidelines [5-7].

Multiple factors can lead to missed reperfusion or reperfusion delays in patients with AMI. Among these factors, failure to perform an ECG upon patient arrival (especially during atypical presentations) and failure to recognize STEMI on the arrival ECG are particularly significant [8]. To address these issues, the ACC/AHA AMI guidelines give a Class 1 recommendation to perform a 12-lead ECG with interpretation within 10 minutes of arrival for all potential AMI patients, including those with atypical or equivalent AMI presentations [5].

**Figure 5:**
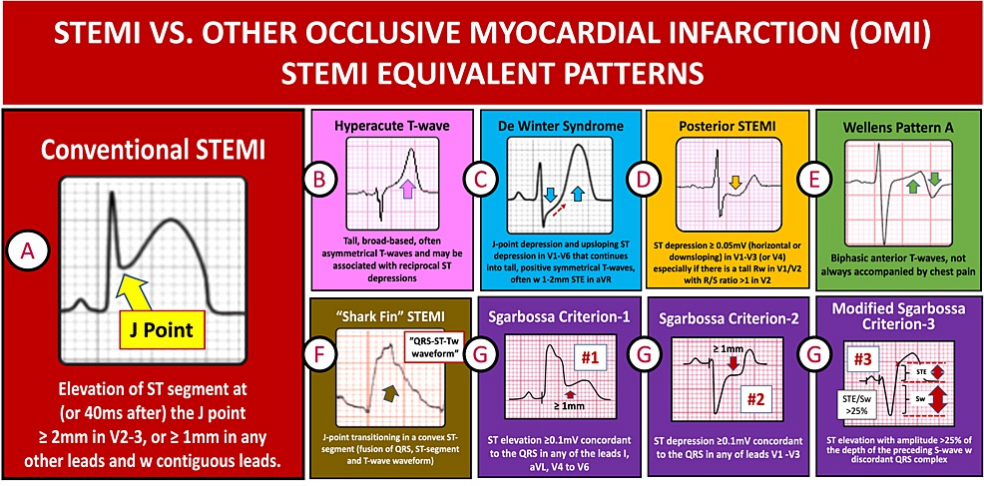
List of OMI STEMI equivalent patterns

The current diagnostic criteria for STEMI are defined as the presence of STE measured after the J-point (the point that connects the end of the QRS wave and the beginning of the ST-segment) of ≥2mm in precordial V2-V3 leads (compared to the isoelectric PQ segment), in two contiguous leads, or ≥ 1mm in any other contiguous leads (Figure 5A) [7].

However, strict STEMI criteria have limited diagnostic accuracy for OMI, missing nearly one-third of OMI cases, leading to incorrect labeling or management as non-STEMI and most importantly depriving patients of emergent reperfusion therapy [9]. Notably, the highest missed rate of OMI is more prevalent in patients with circumflex occlusion, with up to 46% of cases being missed [9]. Moreover, the STEMI criteria have gender and age differences, with a lower STE criterion of ≥ 1.5mm for females in precordial V2-V3 leads and ≥2.5mm for males <40 years of age.

Advances in the recognition of STEMI/OMI patterns have refined diagnostic criteria beyond the current STEMI criteria. Refinements include recognizing early subtle and/or borderline changes, such as with a hyperacute T-wave (Figure 5B) without diagnostic STE, as may be present in the de Winter’s pattern (Figure 5C), which will also have an upsloping ST-segment depression (STD) and STE in aVR [10]. Additionally, other variations include the presence of reciprocal STD on opposing leads, such as non-diagnostic mild STE <1mm in aVL (corresponding to a high lateral OMI) with STD on inferior leads, or the presence of STD in the anterior precordial leads of ≥0.5mm conforming to a posterior (inferobasal) OMI corresponding to reciprocal anterior changes and findings of isolated lead III STE with reciprocal STD in lateral leads (recently described as the Aslanger’s OMI pattern, associated to obstructive multivessel CAD) [11]. Hidden or ECG silent AMI may require additional ECG leads or serial ECG testing for the diagnosis. Guidelines recommend serial ECGs to be performed every 15-30 minutes for patients with persistent AMI clinic findings in the absence of diagnostic STEMI/OMI patterns [5-7]. Additional ECG leads or serial ECG testing may be needed to diagnose a right ventricle OMI (with right-sided precordial leads RV4-RV6, with STE ≥0.5mm) and/or a posterior OMI (Figure 5D) with posterior leads corresponding to V7-V9, with STE ≥0.5mm).

Recognition of reperfusion ST-segment and T-wave changes is usually associated with a large and prompt reduction in STE and T-wave inversions, as may occur after reperfusion therapy or spontaneously, giving a Wellens’ ECG pattern B (Figure 5E), also accompanied by a clinical improvement with chest pain resolution and not to be confused with ongoing STEMI. On the other hand, the presence of ST-elevation on an ECG is not always indicative of AMI, as it can also be observed in several baseline ECG abnormalities and acute conditions, such as pericarditis, takotsubo cardiomyopathy, cor pulmonale, and hyperkalemia. Baseline ECG abnormalities that can confound for STEMI or act as a STEMI mimicker include conditions with repolarization abnormalities, such as early repolarization, left ventricular hypertrophy (LVH), left bundle branch block (LBBB), right ventricular pacing, ventricular aneurysm, and pre-excitation. In fact, a study by Brady et al. investigated the presence of STE (with STEMI criteria) in patients presenting to ED with chest pain and found that only 15% were ultimately discharged with the diagnosis of STEMI. The most frequent cause of STE is LVH [12]. For this reason, it is crucial to identify these ECG confounding conditions (often called mimickers, look-alikes, or pseudoinfarctions) to avoid inappropriate administration of fibrinolytic therapy or the unnecessary activation of catheterization laboratories, resulting in the overuse of resources for non-OMI conditions.

A review of prior ECGs is often helpful in distinguishing a new from a chronic ﬁnding but should not delay the decision for treatment. Patients with conduction disorders, such as LBBB and right ventricular pacing, may also present with abnormal repolarization changes. The presence of STE ≥1 mm concordant with the QRS complex in any lead, along with other Sgarbossa criteria (Figure 5G), such STD of ≥1mm in leads V1-V3 or disproportionate STE (>1mm) compared to the preceding S-wave (>25% of STE/S-wave ratio as the third modified Sgarbossa criteria), may indicate OMI and improve accuracy for OMI diagnosis [13-14].

Authors, such as Aslanger et al., are advocating for a shift from the current STEMI/non-STEMI paradigm to a pathologic substrate of the OMI/non-OMI paradigm to improve diagnostic accuracy. This model aims to increase diagnostic specificity by considering and ruling out mimicking conditions and detecting early minor or distinct OMI patterns to enhance sensitivity [15-16]. Aslanger et al. conducted a prospective study to compare these two paradigms by recruiting a total of 3,000 patients with pre-deﬁned ECG ﬁndings in addition to STEMI criteria. Groups were divided into 1,000 patients with STEMI criteria, 1,000 with non-STEMI criteria, and 1,000 in a control group without AMI. The ﬁnal outcome of the composite ACO endpoint was used to determine diagnostic accuracy [16]. Their results showed that 28% of the patients initially classiﬁed as having non-STEMI were re-classiﬁed as having OMI by the ECG reviewers. This subgroup had a higher frequency of ACO, myocardial damage, and both in-hospital and long-term mortality compared to the non-OMI group. The OMI/non-OMI approach to the ECG demonstrated a superior diagnostic accuracy compared to the STEMI/non-STEMI approach in the prediction of both ACO and long-term mortality. Furthermore, early intervention in patients with OMI-predicting ECGs was associated with lower long-term mortality.

Our current case illustrates another rare OMI pattern, recently described as the SF OMI pattern, which adds to the growing list of uncommon OMI patterns. The SF OMI pattern was named after its characteristic appearance resulting from the QRS fusion to the ST-segment, leading to a distorted terminal R-wave with concomitant STE and T-wave morphology that resembles a “triangular waveform,” lambda-wave, or giant R-wave (Figure 5F). A retrospective analysis of a cohort of 428 consecutive STEMI patients revealed an incidence rate of less than 1.5% in this OMI pattern [1]. The SF OMI pattern was associated with a high risk of mortality from cardiogenic shock or VF [1]. This pattern may occur regardless of the culprit artery; however, the left main and LAD are the most common culprits. Our patient presented with a proximal LAD occlusion, and like in the cohort described above, also developed ventricular arrhythmia and cardiogenic shock. Despite a successful primary PCI to his culprit lesion and hemodynamic support with an Impella CP assist device, the patient died from multiorgan failure and a refractory VT storm. Other non-ischemic conditions, such as myopericarditis and Takotsubo, may also manifest with this electrocardiographic presentation [17-18]. Although no definitive electrophysiological mechanism has been confirmed, changes in sodium channel kinetics and/or dynamics during ischemia have been proposed as plausible explanations [19].

Patients presenting with SF OMI pattern require aggressive treatment with prompt reperfusion due to the increased risk of developing ventricular arrhythmias and hemodynamic consequences from an extensive AMI. A retrospective study by Aizawa et al. found that triangular "coved" ST-T patterns were observed in 61.3% of patients who developed VF, compared to only 9.4% of patients who did not develop VF [20], highlighting a specificity of 90% specificity. However, misinterpretation of this OMI pattern may lead to diagnostic confusion and delayed reperfusion therapy, resulting in a poor outcome. In our case, the patient’s initial presentation with a wide-complex tachycardia was initially interpreted as VT, and management was unsuccessful. Upon careful review of his arrival and follow-up ECGs (Figures 1B, 2), it was observed that the patient’s arrival rhythm was sinus tachycardia as evident by P-waves preceding the QRS waves (marked on his follow-up ECG, more evident upon slowing of his sinus rhythm after amiodarone provided). Additionally, his baseline ECG (Figure 1A) revealed evidence of a chronic RBBB. Finally, the patient had the characteristic features of the SF OMI pattern, such as the fusion of chronic RBBB to STE and T-wave, giving a triangular or SF waveform morphology, with reciprocal changes in inferior leads (Figure 2).

There is a previous case report by Andreou et al., where a patient presenting with an SF OMI pattern (from a left main occlusion) was also confused for VT leading to a delayed reperfusion intervention and a poor outcome [2]. It is, therefore, crucial for frontline physicians to be aware of this relatively new recognized OMI pattern for patients presenting with AMI complaints or post-cardiac arrest ECG to recognize this SF OMI pattern and avoid delays in reperfusion therapy.

## Conclusions

We believe that this SF OMI pattern must be added to the increasing list of OMI patterns that do not meet the rigid STEMI definition criteria, such as early peak T-waves with hyperacute OMI, de Winter’s peak T-wave with anterior precordial STD, Sgarbossa criteria for OMI in LBBB or RV pacing, the Aslanger pattern with inferior subtle non-contiguous STE (not meeting STEMI criteria) and isolated anterior precordial ST depressions with posterior OMI, just to mention a handful of these OMI patterns. The SF OMI ECG pattern may be misdiagnosed as a wide complex tachycardia, as occurred in this case, leading to a delay in STEMI diagnosis. Early recognition of STEMI or OMI patterns is critical to avoid delays in reperfusion therapy and to anticipate and better prepare for a higher-risk OMI pattern that will benefit more from primary PCI reperfusion (instead of fibrinolytic therapy) and to prepare for the potential need for hemodynamic support and ventricular arrhythmia therapy.
